# Cu(II) Removal from Aqueous Samples Using In Situ Functionalized Silica with Novel Synthesized Formazan: Synthesis, Characterization, Kinetic and Isothermal Studies

**DOI:** 10.1021/acsomega.5c05173

**Published:** 2025-08-18

**Authors:** Mine Antep, Yıldız Çelti̇k, Cevher Gündoğdu Hızlıateş̨, Nalan Demi̇r

**Affiliations:** † Department of Chemistry, Faculty of Science, 37508 Dokuz Eylul University, Izmir 35160, Turkey; ‡ The Graduate School of Natural and Applied Science, Dokuz Eylul University, Izmir 35160, Turkey

## Abstract

A novel silica-based sorbent, silica-carbazole-formazan (Si-Carb-Formazan), was synthesized through in situ functionalization with a newly prepared carbazole formazan derivative to remove Cu­(II) ions from aqueous solutions efficiently. The sorbent was characterized using techniques such as FTIR, SEM, TGA, and XPS, which revealed a porous structure with a high surface area and excellent thermal stability. Batch adsorption experiments analyzed the influence of various factors on the sorbent’s performance, demonstrating its high efficiency. Optimal adsorption occurred at a pH of 6.0, with a maximum capacity of 34 mg/g achieved within 15 min using only 5.0 mg of the sorbent. The adsorption process adhered to the Langmuir isotherm model and followed pseudo-second-order kinetics. Also, thermodynamic analysis indicated that the process was exothermic under the studied conditions. The sorbent displayed high selectivity for Cu­(II) ions over other metal ions and retained over 90% of its initial capacity after five regeneration cycles using 0.10 M HCl. These findings highlight the high efficiency and potential of Si-Carb-Formazan as a reusable and cost-effective material for the rapid removal of toxic Cu­(II) ions from contaminated water.

## Graphical abstract

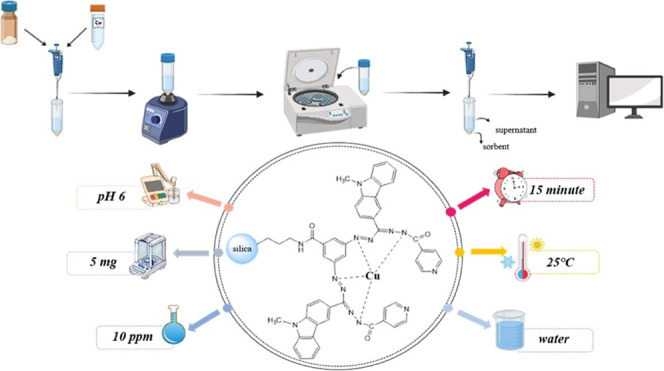

## 1 Introduction

Copper is a trace element of vital importance to humans and is found in various enzymes that play critical roles in important metabolic processes and physiological functions.^1–3^ Additionally, copper is an important strategic material widely used in various industrial processes due to its specific physicochemical properties, such as its catalytic activity in modern organic synthesis.^4,5^ However, from an environmental perspective, copper ions are considered heavy metal pollutants due to their emission and accumulation in the ecological system. Copper is toxic at high concentrations and poses serious risks to humans and the environment due to its bioaccumulative and nonbiodegradable nature. For example, excessive copper can cause conditions such as hemolytic anemia, neurological abnormalities, and even cancer.^4,6^

The increasingly stringent environmental regulations have made developing effective methods for copper recovery from wastewater an urgent necessity. However, traditional wastewater treatment methods often have disadvantages such as high cost, excessive chemical use, and secondary pollution.^7–10^ In removing copper ions (Cu­(II)) , adsorption is one of the most effective methods due to its high efficiency, low cost, ease of operation, and environmental compatibility.^4,11^ While adsorbents such as chitosan, clay, zeolite, biochar, nanocellulose aerogels, and graphene oxide have been developed for this purpose,^12–19^ existing materials are generally inadequate due to low efficiency, limited selectivity, and challenges in disposing of toxic waste. This situation increases the need for multifunctional materials that offer higher uptake capacity, selectivity, and reusability.^4^ On the other hand, mesoporous silica materials stand out due to their high surface area, large pore structure, stability, and modifiability characteristics.^20–23^

Formazans are a significant and unique class of nitrogen-rich organic compounds with an NH—N=C—N=N backbone.^24,25^ They can display thermochromic and photochromic characteristics, making them valuable ligands. Due to their reversible redox conversion to tetrazolium salts, they can also exhibit a range of biological activities^26–28^ and serve as redox indicators. Formazans’ intense hues from π–π* transitions and stable characteristics make them useful as analytical reagents in the spectroscopic detection of various metal ions.^29^ As a result, formazans have a variety of applications, such as indicators, dyes for different materials,^30–32^ catalysts for redox reactions, ligands for adsorption, and ingredients in the optical recording medium and photographic formulations. Because formazans generate stable colored complexes with high molar extinction coefficients, they are also utilized in analytical chemistry for the spectrophotometric detection of many elements.

Creating materials with predefined properties using formazan immobilization onto different matrices, such as cellulose, organic resin, or silica gel, is one of the fields of research in formazan chemistry. These materials could be helpful in heterogeneous catalysis and sorption-spectroscopic and chromatographic analytical techniques. Many sorbents based on silica gels and ion exchangers have been reported,^33,34^ it has been observed that ionic bonds between a base site on the matrix surface and a sulfo group of formazan are typically what keep formazan molecules on the surface. Grafting a previously synthesized formazan is the method described for preparing sorbents that contain formazan. On the other hand, due to the variety of substituents in the hydrazone and diazonium salt, the synthesis of formazans on a solid support using the traditional Ried technique can increase the range of compounds produced.^34^

This study was designed to investigate the equilibrium and kinetics of Cu­(II) removal from aqueous solutions using a newly prepared silica-based sorbent functionalized with a novel formazan derivative, Silica-Carbazole-Formazan (Si-Carb-Formazan). The study was also aimed to examine the impact of various parameters, including pH, adsorption time, metal ion concentration, sample dosage, and the reusability of silica formazan, on its interaction with Cu­(II). This research is crucial for understanding the potential of silica-based sorbents and their formazan derivatives in addressing the urgent need for effective Cu­(II) removal methods.

## 2 Experimental Section

### 2.1 Chemicals and Reagents

Aminopropyltrimethoxysilane, ((C_2_H_5_O)_3_Si­(CH_2_)_3_NH_2_, APTES, 98%), 3-nitrobenzoyl chloride, *N*-3-silicapropyl-3,5-dinitrobenzamide, 9-methyl-9*H*-carbazole-3-carbaldehyde, pyridine-4-carbohydrazide, *N*-3-silicapropyl-3,5-dinitrobenzamide and all chemicals were obtained from Sigma-Aldrich. All the conventional solvents were of Analytical grade and obtained from Merck, Darmstadt, Germany. Copper sulfate (Cu­(SO_4_)_2_·5H_2_O) was obtained from Sigma-Aldrich Chemical Co., Ltd. The Cu­(II) solution was prepared by directly dissolving proper amounts of Cu­(SO_4_)_2_·5H_2_O salt. Sodium hydroxide (NaOH), hydrochloric acid (HCl), nitric acid (HNO_3_), and other reagents were all purchased from Sigma-Aldrich (St. Louis, MO, USA). Ultrapure water used in all experiments was obtained from the Milli-Q ultrapure water purification system (Millipore, Milford, MA, USA).

### 2.2 Preparation of Si-Carb-Formazan Sorbent

#### 2.2.1 Synthesis of Aminopropyl Modified Silica Gel

The mixture of 10 g of dry silica gel (70–230 mesh), 50 mL of dry toluene, and 12 mL of APTES was refluxed for 24 h and filtered after cooling. Then, washing was performed with chloroform, ethanol, and water, respectively. The salmon-colored product was dried in an oven at 60 °C for 6 h.^35^

#### 2.2.2 Synthesis of *N*-3-Silicapropyl-3,5-Dinitrobenzamide Compound

Aminopropyl-modified silica gel (5 g) and triethylenediamine (1.1 mL, 7.7 mmol) were added to a solution of 3,5-dinitrobenzoyl chloride (1.61 g, 7 mmol) in 20 mL chloroform and heated at 50–80 °C for 24 h.^34^

#### 2.2.3 Synthesis of *N*-3-Silicapropyl-3,5-Diaminobenzamide Compound

*N*-3-Silicapropyl-3,5-dinitrobenzamide (5 g), 30 mL of 2-ethoxyethanol, N_2_S_2_O_4_ (9.4 g, 60 mmol), and 30 mL of water were boiled under reflux for 3 h. The product was then left to cool, washed with water, filtered, and dried in the oven.^34^

#### 2.2.4 Synthesis of *N*′-[(9-methyl-9*H*-Carbazole-3-yl)­methylidene]­pyridine-4-carbohydrazide Compound

Into a 100 mL flask, add 9-methyl-9*H*-carbazole-3-carbaldehyde (2 g, 9.57 mmol), pyridine-4-carbohydrazide (1.32 g, 9.57 mmol), ethanol (50 mL), and glacial acetic acid (2 mL) was added. It was then boiled in an oil bath under reflux overnight. After the boiling process was completed, the solvent of the product obtained was removed from the evaporator, then it was crystallized with methanol, and a yellow solid product was received.^36^

#### 2.2.5 Synthesis of 5-(3-silicapropylcarbamoyl-5-(2-isonicothionoylhydrazano)­(9-methyl-9*H*-carbazole-3-ylmethyl)­diazenyl)­phenyl)-1-isonicotinoyl-3-(9-methyl-9*H*-carbazole-3-yl)­formazan Compound

*N*-3-Silicapropyl-3,5-dinitrobenzamide (5 g),H_2_O (2 mL), and HCl (0.72 mL, 23 mmol) were stirred at −5 °C. NaNO_2_ (0.48 g, 6.9 mmol) in H_2_O (1 mL) was dissolved at −5 °C for 40 min. After the addition of *N*-((9-methyl-9*H*-carbazol-3-yl) methylene isonicotinohydrazide (0.88 g, 2.6 mmol) and NaOH (0.0336 g, 0.84 mol) in 30 mL of ethanol, the mixture was stirred for 2 h at −5 °C and for 40 min at room temperature. The filtered product was washed in ethanol and dried at 75 °C.^34^

### 2.3 General Scheme and Synthesis Plan of the Study

In the synthesis plan of this study, the silica-3,5-dinitrobenzamide compound was obtained by acylation of 3-aminopropyl silica gel and 3-nitrobenzoyl chloride. Then, the nitro group was reduced in the presence of sodium dithionite and 2-ethoxyethanol to form the silica-3,5-diamino-*N*-butylbenzamide structure. The resulting structure formed the diazonium salt by an aromatic electrophilic substitution reaction. Finally, the carbazole phenyl hydrazone group is bonded to the resulting structure.

Consequently, silica-1-(3-butyl carbamoyl-5­(((2-isonicotinoylhydrazinilidene)­(9-phenyl-9*H*-carbazole-3-yl)­methyl)­diazenyl)­phenyl)-5isonicotinoyl-3-(9-phenyl-9*H*-carbazole-2-yl) formazan sorbent (Si-Carb-Formazan) was synthesized in this study (Figure 1).

**Figure 1:**
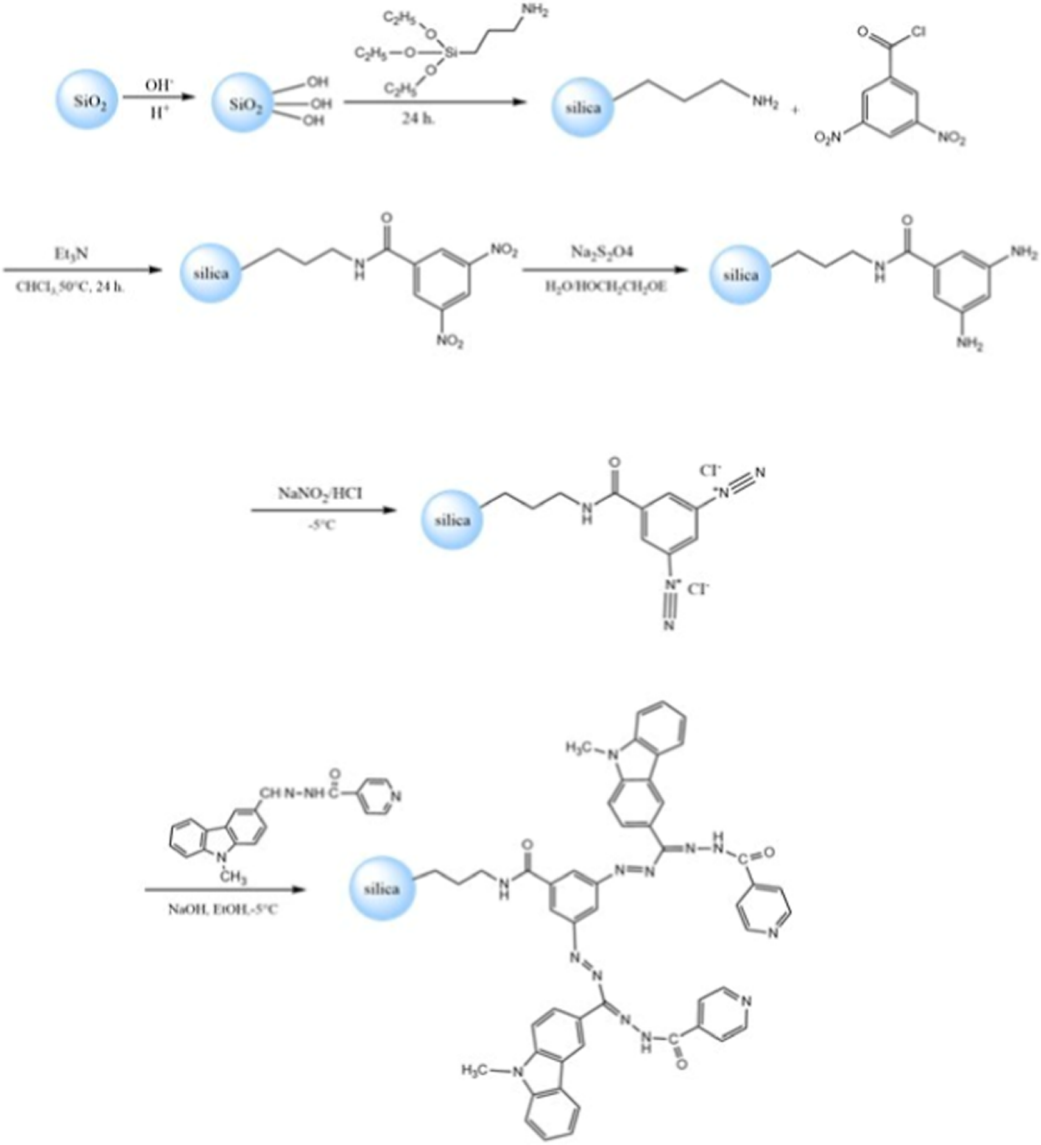
In situ functionalized silica.

### 2.4 Removal of Cu­(II) with Si-Carb-formazan Sorbent

Batch adsorption experiments were conducted under controlled conditions, including variables such as initial pH (4–7), adsorbent amount (1–10 mg), initial metal ion concentration (1–500 mg/L of Cu­(NO_3_)_2_·3H_2_O), contact time (5–30 min), and temperature (15–45 °C) by stirring at 150 rpm. The mixture was then centrifuged at 10,000 rpm using Rotina 380 R. Concentrated HCl/NaOH solution was dropped into the Cu­(II) solution to bring its pH to the desired level. The supernatant was analyzed with a flame atomic absorption spectrophotometer (FAAS) to determine the amount of copper ions removed. A PerkinElmer AAnalyst 700 FAAS was used to determine the concentrations of Cu­(II) in an air-acetylene flame.

The adsorption capacity *Q* (mg/g) of the Si-Carb-Formazan sorbent was calculated using eq 1:

1 $$Q=\left(C_{0}-C\right)\times V/m$$

where *C* (mg/L) is the ion concentration determined by AAS, *V* (L) is the volume of solution, *C*_0_ (mg/L) is the initial ion concentration, and *m* (g) is the amount of adsorbent.

### 2.5 Desorption and Reuseability Experiments

The desorption of Cu­(II) ions was examined in 0.1 M HNO_3_, 0.1 M HCl, and 0.1 M NaOH solutions. The Cu­(II)-loaded Si-Carb Formazan sorbent was placed in the desorption medium and stirred at room temperature for 15 min. FAAS determined the final concentration of Cu­(II) ions in the aqueous phase. The desorption rate was calculated based on the amount of Cu­(II) adsorbed onto the sorbent and the final concentration of Cu­(II) in the desorption medium, utilizing the following eq 2.

2 $$Heavy\;metal\;desorption\left(\%\right)=\frac{C_{0}-C_{i}}{C_{0}}\times 100$$

where *C*_0_ is the concentration of the metal bound to the adsorbent before the desorption experiment (mg/L) and *C*_i_ is the concentration of the metal remaining on the adsorbent after the experimental analysis (mg/L).

This equation allows for the calculation of the percentage of metal ions that have been desorbed. When the adsorption–desorption cycles are repeated, this method can determine the desorption rate in each cycle. This way, the reusability of the Si-Carb Formazan sorbent can be evaluated. To evaluate the reusability of the Si-Carb Formazan sorbent, adsorption–desorption cycles were repeated five times using the same sorbent.

### 2.6 Kinetic, Isothermal, and Thermodynamic Studies

The adsorption isotherm provides the most significant information regarding the distribution of adsorbed molecules between the solid and the liquid when the adsorption process reaches equilibrium. In this study, the Langmuir, Freundlich, Temkin, and Dubinin–Radushkevich isotherms were employed to evaluate the interactions between the adsorbate and the adsorbent in equilibrium.

The Langmuir isotherm describes monolayer adsorption on a surface. There is no migration of adsorbed species between layers of an adsorbent with an infinite number of adsorption sites. The linear equation for the Langmuir isotherm is expressed as

3 $$\frac{C_{e}}{q_{e}}=\frac{1}{Lq_{m}}+\frac{C_{e}}{q_{m}}$$

where *q*_e_ is the amount of adsorbed Cu­(II) per unit weight of adsorbent at equilibrium (mg/g), *C*_e_ is the equilibrium concentration of Cu­(II) in the solution (mg/L), *q*_m_ indicates the maximum adsorption capacity for monolayer adsorption (mg/g), and *L* is related to the adsorption energy (L/mg). When *C*_e_/*q*_e_ is plotted against *C*_e_, the slope of the resulting equation yields 1/*q*_m_, and the intercept is 1/(*Lq*_m_).^37^

The equilibrium constant underlying the Langmuir isotherm is defined as a dimensionless constant designated *R*_L_. The following equation (eq 4) was suggested by Weber and Chakravorti (1974):^37^

4 $$R_{L}=1/\left({1+LC}_{0}\right)$$

where *L* is the Langmuir constant and *C*_0_ is the maximum initial concentration of Cu­(II) (mg/L).^37^ The presence of *R*_L_ values ranging between 0 and 1 in discontinuous system adsorption indicates that the adsorption process occurs spontaneously.

The Freundlich model is predicated on adsorption onto heterogeneous surfaces or surfaces with varying affinities. It is posited that regions of stronger binding are filled first, while the binding strength diminishes as the degree of occupancy of the binding sites increases. The linear form of the Freundlich eq 5 is as follows

5 $$\log q_{e}=\log K_{f}+n_{f}\;\log C_{e}$$

The Freundlich constant *K*_f_ (L/g) denotes the adsorption capacity, while *n*_f_ represents the heterogeneity factor in the adsorption process. The plot of log *q*_e_ against log *C*_e_ gives *n*_f_ as the slope and log *K*_f_ as the intercept.^38^ The *n*_f_ value, which lies between 0 and 1, serves as an indicator of surface heterogeneity. A value of *n*_f_ < 1 aligns with normal Langmuir isotherm behavior, while *n*_f_ > 1 suggests cooperative adsorption.^39^

The Temkin isotherm incorporates a factor that elucidates the relationships between the adsorbent and adsorbate. Adsorption is characterized by a regular distribution of binding energies up to certain maximum values. The Temkin isotherm is expressed as follows eq 6:

6 $$q_{e}=\left(\frac{RT}{b_{T}}\right)\;\ln\left({AC}_{e}\right)$$

In this equation, *RT*/*b*_T_ = *B* (J/mol) is the Temkin constant, which is related to the adsorption heat; *A* (L/g) is the equilibrium binding constant, which is dependent on the maximum binding energy; *R* = 8.314 J/mol K is the universal gas constant; and *T* (K) is the absolute temperature of the solution.^40^

To calculate the average free energy of adsorption, the Dubinin–Radushkevich (D-R) isotherm (eq 7) is utilized:

7 $${\ln q}_{e}={\ln X}_{m}-{\beta \varepsilon }^{2}$$

where β is a constant associated with the adsorption energy (mol^2^/J^2^), *X*_m_ is the DR monolayer capacity (mg/g), and *q*_e_ is the quantity of adsorbed Cu­(II). The Polanyi potential, ε, is calculated using eq 8:

8 $$\varepsilon =RT\;\ln\left(1+1/C_{e}\right)$$

The parameters β and *X*_m_ are derived from the slope and intercept, respectively, of the graph of ln *q*_e_ versus ε^2^.^41^ The average free energy of adsorption, *E* (kJ/mol), is calculated using eq 9 with the determined β value:

9 $$E=\frac{1}{{2\beta }^{1/2}}$$

To determine the adsorption rate of Cu­(II), adsorption kinetics was employed, enabling control over the equilibrium time. The pseudo-first-order kinetic model is widely used to evaluate adsorption kinetics. This model, defined by Lagergren (1898), is expressed by eq 10:^42^

10 $$\ln\left(q_{e}-q_{t}\right)=\ln q_{e}-k_{1}t$$

where *q*_e_ and *q*_*t*_ (mg/g) are the quantities of adsorbed Cu­(II) at equilibrium and at time *t*, respectively, and *k*_1_ (1/h) is the adsorption rate constant. When the natural logarithm of *q*_e_ – *q*_*t*_ is plotted against time, the slope obtained from the resulting linear equation yields *k*_1_, while the intercept provides the value of ln *q*_e_.^42^

The pseudo-second-order equation based on equilibrium adsorption is represented as follows:

11 $$\frac{t}{q_{t}}=\frac{1}{k_{2}{q_{e}}^{2}}+\frac{t}{q_{e}}$$

in which *k* ^2^ (g/mg h) is the rate constant. The slope of the linear equation derived from plotting *t*/*q*_*t*_ against time gives 1/*q*_e_, while the intercept corresponds to 1/(*k*_2_ *q*_e_^2^). This method generally predicts the behavior across the entire range of adsorption.^43^

To analyze the applicability of adsorption data, the Elovich equation is utilized:

12 $$q_{t}=\frac{1}{\beta }\;\ln\left(\alpha \beta \right)+\frac{1}{\beta }\;\ln\left(t\right)$$

in which α (mg/g h) is the initial adsorption rate and β (g/mg) is the surface diffusion coefficient. The parameters 1/β and 1/β ln­(αβ) correspond to the slope and intercept obtained from plotting *q*_*t*_ against ln­(*t*).^44^ The value of 1/β indicates the number of suitable regions for adsorption, while 1/β ln­(αβ) expresses the amount of adsorption when ln *t* is equal to zero. This value facilitates understanding the adsorption behavior of the initial stage.^45^

The aforementioned kinetic models do not sufficiently explain the diffusion mechanism, and therefore, the intraparticle diffusion model proposed by Weber and Chakravorti (1974) is employed.^37^ In most adsorption processes, the relationship is characterized by a functional dependency that varies in proportion to *t* ^1/2^ rather than simply the contact time. The theoretical equation is as follows:

13 $$q_{t}=k_{pi}t^{1/2}+C_{i}$$

the rate parameter of stage *i*, denoted by *k*_p*i*_ (mg/g h^1/2^), is determined from the slope of the linear equation derived from plotting *q*_*t*_ against *t* ^1/2^. The intercept of this equation provides insight regarding the thickness of the boundary layer. For instance, larger values of *C*_*i*_ are indicative of a greater boundary layer effect.^37^

To investigate the thermodynamic behavior of Cu­(II) adsorption on the Si-Carb-Formazan sorbent, thermodynamic parameters were determined at four distinct temperatures (288, 298, 308, and 313 K). The thermodynamic parameters were calculated using eqs 14–16:

14 $$K_{d}=\frac{q_{e}}{C_{e}}$$

15 $${\Delta G}^{{}^{\circ}}=-RT\;\ln K_{d}$$

16 $${\ln K}_{d}=-\frac{{\Delta H}^{{}^{\circ}}}{RT}+\frac{{\Delta S}^{{}^{\circ}}}{R}$$

where *K*_d_ is the thermodynamic equilibrium constant, *T* (K) is the absolute temperature, Δ*S*° (J/mol K) is the standard entropy change of the system, Δ*H*° (kJ/mol) is the standard enthalpy change, and Δ*G*° (kJ/mol) is the standard Gibbs free energy change.

### 2.7 Effect of Interferences

The effect of interference ions on the adsorption of Cu­(II) was performed by mixing 5.0 mg of the Si-Carb-Formazan sorbent with 10 mL of the Cd­(II), Zn­(II), Fe­(III), Pb­(II) solutions at a concentration of 10 mg/L. FAAS analyzed the concentrations of heavy metal ions in the solution.

### 2.8 Real Sample Studies

Water samples were provided and gathered in 100 mL glass bottles, including drinking water from local sources and tap water from the laboratory. Water samples were filtered through filter paper having pores that were 0.45 μm in size, and the pH of the samples was adjusted to 6.0. Before use, they were stored at 4 °C in the dark. For real sample analysis, 1.0, 2.0, and 5.0 mg L Cu­(II) were added to the water samples. The optimized removal procedure was used to test the removal effectiveness of Si-Carb-Formazan for Cu­(II) in tap water and bottled drinking water. All the experiments were repeated three times.

The error bars shown in the graph were calculated to indicate the variability of the experimental data. It is important to note that for each group of measurements, the mean values, which serve as the anchor for our understanding, were presented together with the standard error of the mean, represented as ± the standard error of the mean. The standard error of the mean was calculated by dividing the standard deviation of replicate measurements by the square root of the number of replicates. This method is commonly used to assess the reliability of the data and to evaluate the statistical significance of differences between means.

## 3 Results and Discussion

### 3.1 Characterization of Si-Carb-Formazan

The Fourier Transform Infrared (FT-IR) spectra of the synthesized ligands and sorbent were recorded with the standard KBr discs on a PerkinElmer Spectrum 100 FT-IR spectrometer (PerkinElmer, Waltham, MA, USA). When the FT-IR spectrum of the Si-Carb-Formazan sorbent was examined, the amide NH, the amide C=O, N=N, and aromatic protons were observed at 3465 cm^–1^, 1677 cm^–1^, 1595 cm^–1^, and 1498–1470 cm^–1^ range, respectively. When the spectra were compared, it was determined that due to the interaction of Cu^2+^ ions with the lone pairs of electrons on nitrogen and amide NH groups, there was an expansion in the amide NH and C=O bands, and the C=O band shifted to 1639 cm^–1^. The N=N peak at 1595 cm^–1^ was not observed due to the broadened amide carbonyl band (Figure 2).

**Figure 2:**
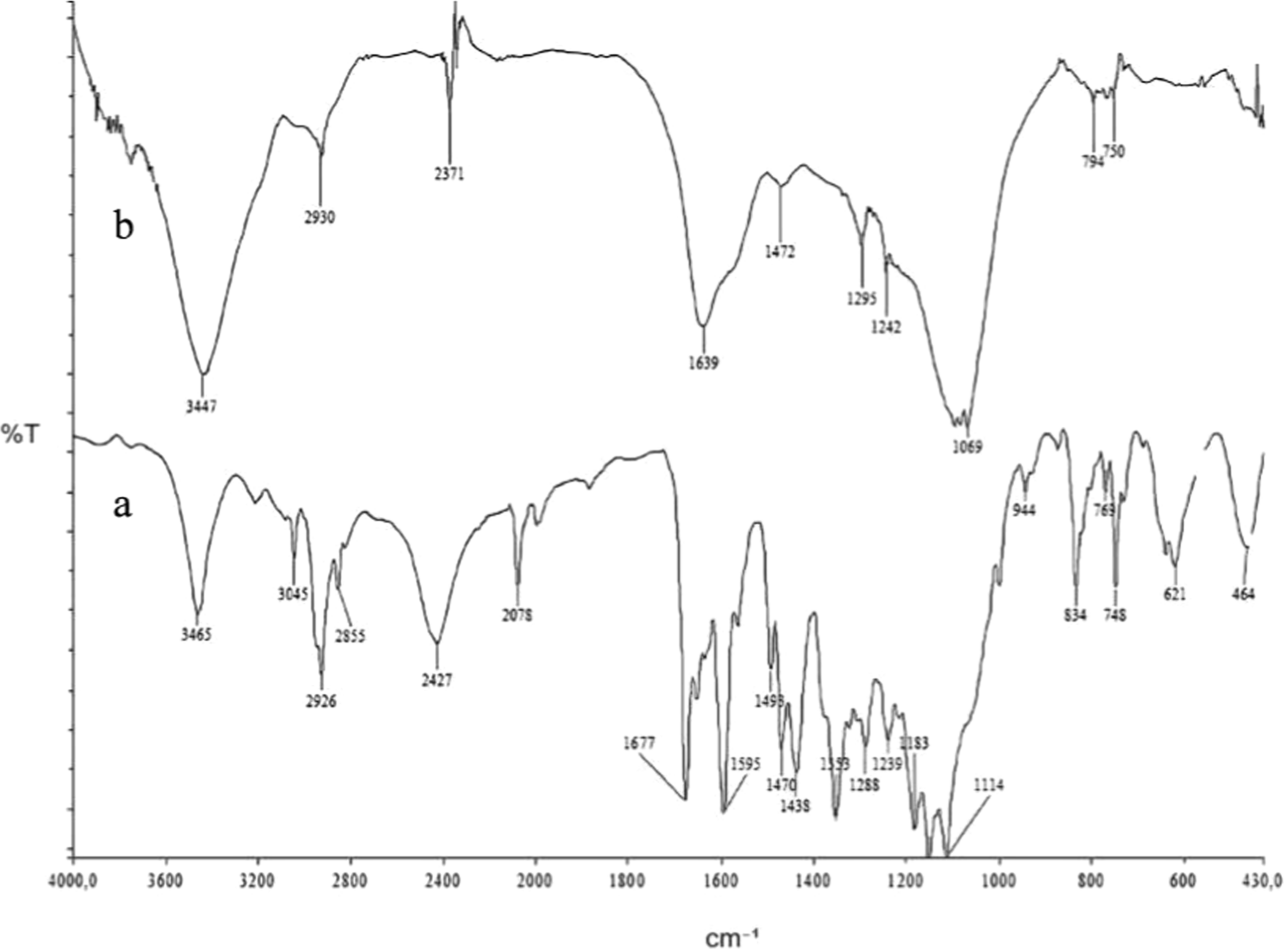
FT-IR spectra of Si-Carb-Formazan (a) before and (b) after Cu adsorption.

The surface morphology of the silica formazan was visualized using a ZEISS Scanning Electron Microscope (SEM) (Carl ZEISS Microscopy, Oberkochen, Germany) after coating with a gold film at an acceleration voltage of 3 kV. Figure 3 shows the SEM images of the Si-Carb-Formazan sorbent. The sorbent exhibited a highly porous morphology. The porous structure increases the surface area of the material, which means that the developed materials can function as suitable sorbents, offering high accessibility to target molecules.

**Figure 3:**
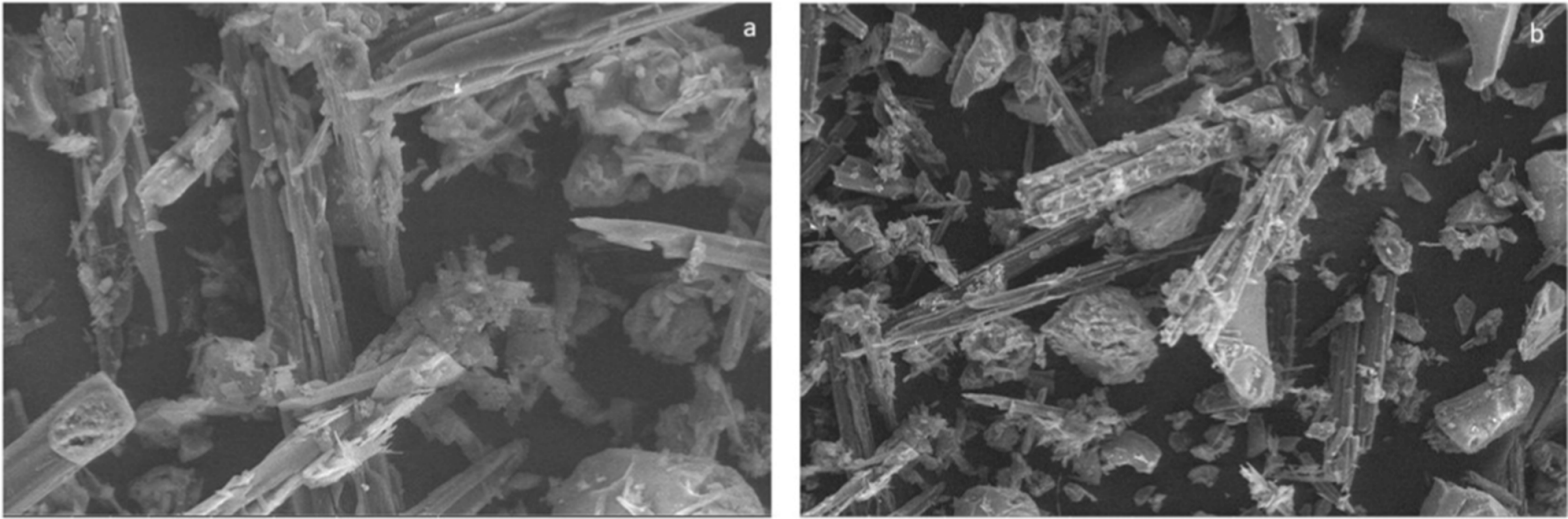
SEM images of Si-Carb-Formazan: (a) 2500× and 50 μm, (b) 1000× and 100 μm.

The thermal properties of the silica formazan were investigated using a PerkinElmer STA 6000 thermogravimetric/differential thermal analyzer (TG/DTA) covering a temperature range of 30–400 °C at a heating rate of 10 °C/min under a nitrogen atmosphere. The thermal decomposition profile of the Si-Carb-Formazan sorbent can be divided into three distinct stages, as illustrated in Figure 4. The initial weight loss, occurring around 100 °C, is attributed to the desorption of physically adsorbed water molecules from the nanosorbent surface. The second stage of mass loss occurs between 100 and 223 °C, with an approximate weight reduction of 1.28% observed at 223 °C. The final stage begins at 350 °C and extends to 423 °C, indicating further material degradation. These findings suggest that the Si-Carb-Formazan sorbent exhibits considerable thermal stability.

**Figure 4:**
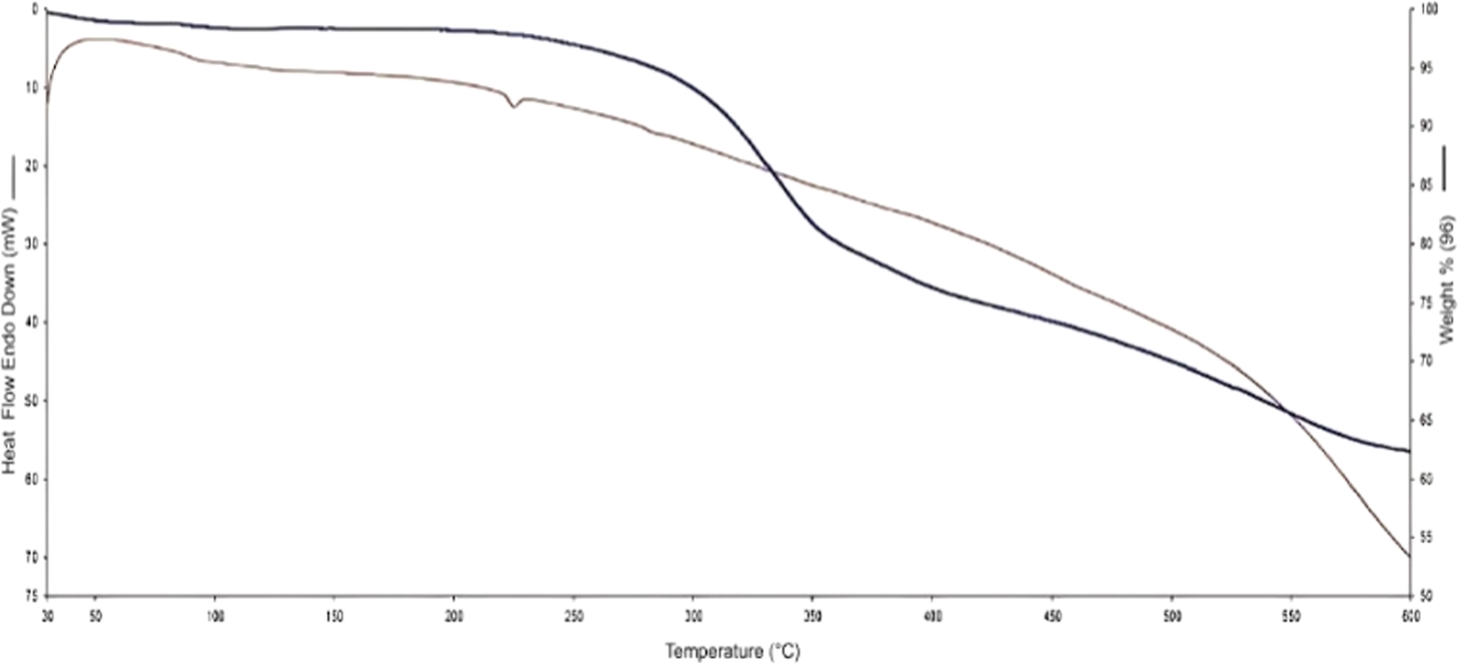
TGA curve of Si-Carb-Formazan.

Thermo Scientific K-Alpha (Waltham, Massachusetts, U.S.) was used for XPS analysis. The XPS survey spectrum presented in Figure 5 was employed to analyze the surface elemental composition of the Si-Carb-Formazan sorbent. The presence of oxygen, nitrogen, silisium, and carbon was confirmed by their characteristic binding energies observed at 532.28 eV, 399.4 eV, 103.07, and 284.87 eV, respectively, in the CS of the sample. Chemical composition and the bonds between the atoms given in Table 1 confirm the possible structure of the developed sorbent.

**Figure 5:**
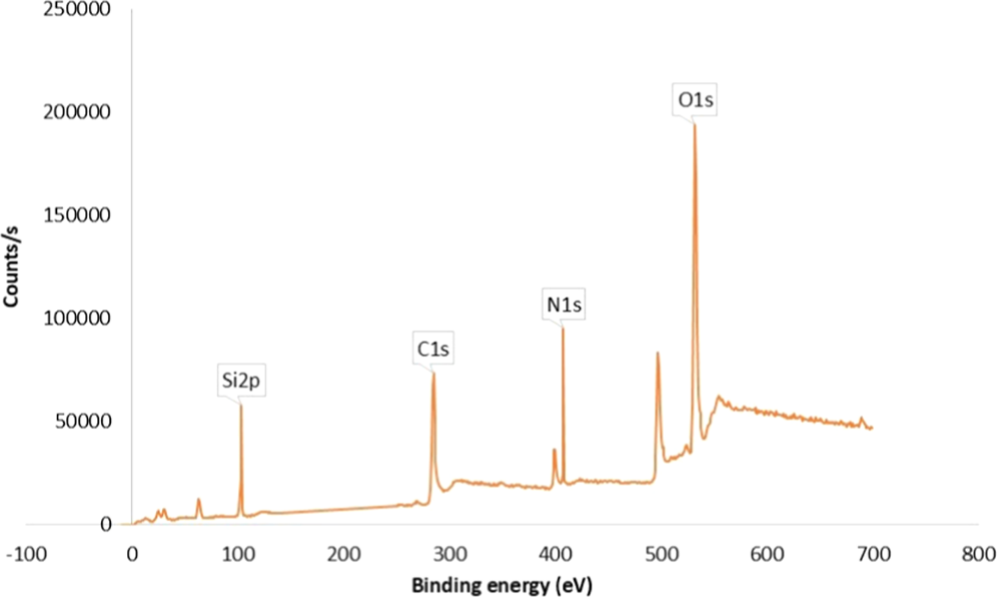
XPS survey spectra of the Si-Carb-Formazan.

**Table 1:** XPS Quantitative Analysis of the Si-Carb-Formazan

element	wt %	chemical composition	spectral region	details
C	26.8	carbon	C 1s	C–C or C–H
		Si carbide	Si 2p	SiC
			C 1s	carbide
Si	24.36	Si oxide	Si 2p	SiO_2_
			O 1s	SiO_2_
N	15.19	Si nitride	Si 2p	Si_3_N_4_
			N 1s	Si_3_N_4_
O	33.65	Si oxynitride	N 1s	SiO_*x*_N_*y*_, *x* > *y*
			N 1s	SiO_*x*_N_*y*_, *y* ≥ *x*

### 3.2 Effect of pH

The pH of the solution significantly impacts the adsorption process; therefore, investigating the pH is an indispensable aspect of enhancing adsorption efficiency. In aqueous solutions, copper ions can exist in various forms, including Cu^2+^, Cu­(OH)^+^, Cu­(OH)_2_, Cu­(OH)_3_, and Cu­(OH)_4_. Notably, the precipitation of Cu^2+^ as Cu­(OH)_2_ when the pH exceeds 7.0 does not accurately account for the adsorption phenomenon. Consequently, the effect of pH on Cu­(II) adsorption onto the Si-Carb-Formazan sorbent was studied within the pH range of 4–7. As illustrated in Figure 6a, the adsorption capacity of the sorbent exhibited an increasing trend with rising pH, followed by a decline, reaching a maximum value of 3.1 mg/g at pH 6. At low pH values, protonation of the amino groups on the sorbent surface results in a positively charged adsorbent, leading to electrostatic repulsion with Cu­(II).^1^ With increasing pH, the adsorption capacity of the sorbent for Cu­(II) increased, peaking at pH 6.0. This increase may be attributed to a reduction in protonation, an enhancement in chelation, and the reinforcement of active sites for Cu­(II) binding, thereby facilitating adsorption.^46^ However, once the pH exceeded 6.0, the adsorption capacity (*Q*_e_) likely began to decrease gradually due to the competitive adsorption of OH-.^47^ Overall, this experiment achieved the optimum adsorption capacity and Cu­(II) removal using the Si-Carb-Formazan sorbent at pH 6, with values of 3.1 mg/g and 44.3%, respectively.

**Figure 6:**
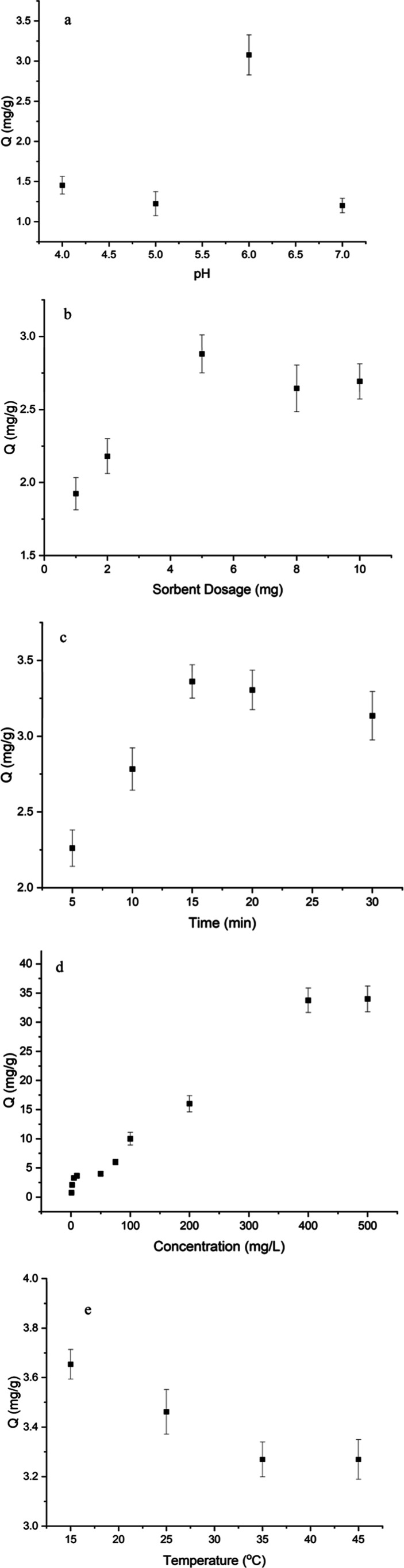
Effect of (a) of pH (0.005 g sorbent, 10 mg/L Cu­(II), 25 °C, 15 min); (b) sorbent dosage (pH 6, 10 mg/L Cu­(II), 25 °C, 15 min); (c) contact time (pH 6, 0.005 g sorbent, 10 mg/L Cu­(II), 25 °C); (d) initial concentration (pH 6, 0.005 g sorbent, 25 °C, 15 min); (e) temperature (pH 6, 0.005 g sorbent, 10 mg/L Cu­(II), 15 min) on the adsorption of Cu onto Si-Carb-Formazan.

### 3.3 Effect of Sorbent Amount

To enhance the interaction between metal ions in the solution and the adsorption sites of the sorbent, it is crucial to determine the optimal amount of sorbent required. The adsorption performance was evaluated by adding varying amounts of Si-Carb-Formazan (1.0, 2.0, 5.0, 8.0, and 10.0 mg) to a solution of Cu­(II) at a concentration of 10 mg/L and a volume of 5 mL, followed by agitation at 25 °C for 15 min. As illustrated in Figure 6b, increasing the dosage of Si-Carb-Formazan from 1.0 mg to 5.0 mg increased Cu­(II) removal efficiency by about 1.5 to 3.0 mg/g. This enhancement can be attributed to the increased availability of Cu­(II) adsorption sites due to the higher dosage. However, the adsorption capacity of Si-Carb-Formazan for Cu­(II) showed a slight decrease with increasing dosage. This reduction may be associated with the partial aggregation or overlap of adsorption sites at higher dosages, leading to a decrease in the number of effective adsorption sites and ultimately resulting in a reduction in adsorption capacity.^1,48^ Considering the adsorption capacity and removal efficiency, 5.0 mg of Si-Carb-Formazan was selected for subsequent experiments.

### 3.4 Effect of Initial Concentration

During the adsorption process, the initial concentration of Cu­(II) in the solution is crucial in overcoming the resistance to mass transfer of the metal between the solution and the sorbent. Consequently, the initial concentration of Cu­(II) plays a critical role in the adsorption process.^49^ To investigate this, Cu­(II) solution with concentrations varying between 50 mg/L and 500 mg/L was prepared in 5 mL, with the pH adjusted to 6.0. Subsequently, 5.0 mg of Si-Carb-Formazan was added to the Cu­(II) solution, stirring until equilibrium was reached. The highest initial concentration was limited to 500 mg/L because Cu ions precipitated as hydroxides above 500 mg/L. As illustrated in Figure 6c, an increase in the initial Cu­(II) concentration from 50 to 400 mg/L resulted in an enhanced adsorption capacity of the sorbent. At the same time, no significant changes were observed at initial Cu­(II) concentrations above this range. Three potential explanations for these results can be proposed^47,50^ (i) A higher initial Cu­(II) concentration increases the driving force to overcome the mass transfer resistance between the liquid and solid phases, thus enhancing the likelihood of collisions between Cu­(II) and the adsorption sites on the Si-Carb-Formazan surface, (ii) an elevated initial Cu­(II) concentration may lead to a more intense interaction between Cu­(II) and Si-Carb-Formazan, thereby improving the efficacy of the adsorption sites on the Si-Carb-Formazan surface, (iii) at Cu­(II) concentrations exceeding 400 mg/L, all adsorption sites on the sorbent surface may have reached saturation. In conclusion, under these conditions, the maximum adsorption capacity of the Si-Carb-Formazan sorbent was determined to be 34 mg/g. Table 1).

### 3.5 Isotherm Studies

The isotherm parameters were calculated based on the results obtained from examining the effect of the initial Cu­(II) concentration on the adsorption onto the sorbent surface, and the results are presented in Table 2.

**Table 2:** Adsorption Isotherm Parameters for Cu

Langmuir isotherm					Freundlich isotherm		
*q*_e_ (mg/g)	*L* (L/mg)	*q*_m_ (mg/g)	*R*_L_	*R* ^2^	*n*_f_	*K*_f_ (mg/g)	*R* ^2^
34.00	0.077	38.46	0.025	0.9908	0.4902	0.176	0.9412

Temkin isotherm				Dubinin-Redushkevich isotherm			
*A* (L/g)	*b*_T_	*B*	*R* ^2^	*Β* (mol^2^/J^2^)	*X*_m_ (mg/g)	*E* (kJ/mol)	*R* ^2^
1.368	1327.46	1.8664	0.9484	2 × 10–8	26.36	5.00	0.9845

As seen from Table 2, the correlation obtained from the fitting of the Langmuir model is better than the fit using either the Freundlich or Temkin models. The fact that the *Q*_m_ value obtained from the Langmuir model is close to the experimentally determined *Q*_e_ value also supports this situation. *R*_L_ value (0.025) derived from the Langmuir isotherm data, being between 0 and 1, indicates that the adsorption process is favorable. On the other hand, the fact that the *n*_f_ value (0.4902) from the Freundlich isotherm data is less than 1 indicates that the adsorption of Cu­(II) conforms to the standard Langmuir isotherm, which assumes that adsorption occurs on a homogeneous surface, where a finite number of identical sites are available. Furthermore, the Dubinin–Radushkevich single-layer capacity value is calculated to be quite close to the maximum adsorption capacity (*X*_m_ = 26.36 mg/g). Energy values ranging between 1 and 8 kJ/mol indicate that the sorption is due to physical interactions between adsorbent and adsorbate.^51^ The value of *E* (5.0 kJ/mol) calculated according to the Dubinin–Radushkevich isotherm equation indicates that physisorption due to weak van der Waals forces plays a significant role in adsorption.

### 3.6 Effect of Time

The evaluation of the adsorption behavior of adsorbents is contingent mainly upon contact time. The adsorption performance of the Si-Carb-Formazan sorbent concerning Cu­(II) was investigated by adding 5.0 mg of Si-Carb-Formazan to a solution containing 10 mg/L of Cu­(II) at a temperature of 25 °C across various contact times ranging from 5 to 30 min. Analysis of the results presented in Figure 6d revealed a substantial initial increase in the adsorption capacity of Cu­(II), attributable to the presence of multiple active sites. However, this increase gradually diminished over time, and no significant change in adsorption capacity was observed beyond 15 min. These findings established a contact time of 15 min for subsequent experiments to determine the adsorption equilibrium.

### 3.7 Kinetic Studies

The dynamic experimental data were analyzed using the pseudo-first-order and pseudo-second-order kinetic models and the intraparticle diffusion models. The adsorption kinetics, which determine the equilibrium time, were assessed based on the Cu­(II) adsorption data, with the results summarized in Table 3. The data clearly show that the correlation coefficient of the pseudo-second-order model surpasses those of the other models, identifying it as the most suitable model for describing the adsorption of Cu­(II).

**Table 3:** Adsorption Kinetic Parameters for Cu­(II)

Pseudo-First-Order Kinetic Model			
*q*_e,exp_ (mg/g)	*q*_e,cal_ (mg/g)	*k*_1_ (1/h)	*R* ^2^
3.13	2.55	9.49	0.9886

Pseudo-Second-Order Kinetic Model				
*q*_e,cal_ (mg/g)	*k*_2_ (g/mg h)	*t*_1/2_ (min)	*h*_0,2_ (mg/g min)	*R* ^2^
3.93	3.95	3.84	62.50	0.9992

Elovich			
1/β ln(αβ) (mg/g)	α (mg/g h)	β (g/mg)	*R* ^2^
4.16	177.26	1.30	0.9978

Intraparticle Diffusion Model		
*k*_p*i*_ (mg/g h^1/2^)	*C*_*i*_	*R* ^2^
0.78	0.96	0.9444

Furthermore, the experimental *q*_e_ value aligns closely with the calculated *q*_e_ value using the pseudo-second-order model, indicating that the rate-limiting step involves chemisorption or chemical adsorption. This process likely includes valence forces through electron exchange or sharing between adsorbents and adsorbates. Based on this model, the rate constant was determined to be 3.95 g/mg h.

Additionally, applying the intraparticle diffusion model revealed a dual-phase adsorption mechanism. The initial phase is characterized by rapid Cu­(II) adsorption on the outer surface of the Si-Carb-Formazan sorbent, driven by strong electrostatic interactions. The subsequent phase involves a slower adsorption step that progresses until equilibrium is achieved, where all available adsorption sites on the sorbents are saturated with Cu­(II).

The linearity of the *t*/*q*_*t*_ versus *t* plot, along with a nonzero intercept, confirms the involvement of intraparticle diffusion in the process. However, the results suggest that intraparticle diffusion is not the sole rate-determining step. Instead, the adsorption process involves a combination of surface adsorption and intraparticle diffusion.

The adsorption kinetics of Cu­(II) onto the Si-Carb-Formazan sorbent conformed to the pseudo-second-order model, exhibiting a high correlation coefficient (*R* ^2^ = 0.9992), which is commonly interpreted as indicative of chemisorption. Nevertheless, it is recognized that pseudo-second-order kinetics can also effectively describe systems predominantly governed by physisorption, particularly in cases involving surface heterogeneity or the presence of multiple adsorption sites.^43^

To gain a deeper understanding of the adsorption mechanism, isotherm analysis and Dubinin–Radushkevich (D–R) model parameters given above were evaluated as follows:

- The D–R isotherm produced a mean free energy (*E*) value of 5.00 kJ/mol, which is well within the characteristic range of physisorption (*E* < 8 kJ/mol) and significantly below the threshold typically associated with chemisorption (*E* > 20 kJ/mol).
- The Freundlich constant (*n*_f_ = 0.4902) suggests a favorable yet relatively weak interaction between the adsorbate and the adsorbent.
- Additionally, the high desorption efficiency observed with 0.10 M HCl and the retention of more than 90% of adsorption capacity after three consecutive cycles further support a reversible physical adsorption mechanism.

Collectively, these findings indicate that physisorption is the predominant mechanism, likely governed by surface interactions such as van der Waals forces or electrostatic attractions. The excellent fit to the pseudo-second-order kinetic model is more plausibly attributed to the heterogeneous nature of the sorbent surface, rather than genuine chemisorptive behavior.

### 3.8 Effect of Temperature

The efficiency of Cu­(II) removal is influenced by temperature in different ways, depending on the adsorption method used. Higher temperatures can improve adsorption by increasing ion diffusion, but may also decrease adsorption if the process is exothermic. Cu­(II) adsorption onto Si-Carb-Formazan was investigated across a 15–45 °C temperature range. As illustrated in Figure 6e, the adsorption capacity of the sorbent decreased significantly with increasing temperature. This observation aligns with the exothermic nature of the adsorption process, consistent with the well-established principle that elevated temperatures often reduce adsorption efficiency in exothermic systems. The decline in performance may be attributed to weakened interactions between Cu­(II) ions and the adsorbent’s active sites at higher temperatures. Specifically, thermal energy likely disrupts the binding forces (e.g., electrostatic attraction, complexation, or van der Waals interactions), reducing the adsorption capacity.

### 3.9 Thermodynamic Studies

To elucidate the thermodynamic characteristics of Cu­(II) adsorption onto the Si-Carb-Formazan sorbent, key thermodynamic parameters were systematically evaluated at four different temperatures: 288, 298, 308, and 313 K. The results were given in Table 4.

**Table 4:** Adsorption Thermodynamic Parameters for Cu

		Δ*G*° (kJ/mol)			
Δ*H*° (kJ/mol)	Δ*S*° (J/mol K)	288 K	298 K	308 K	313 K
–3.00	–21.08	3.05	3.29	3.55	3.66

Thermodynamic analysis revealed a negative enthalpy change (Δ*H*° = −3.00 kJ/mol), indicating that the adsorption of Cu­(II) onto Si-Carb-Formazan sorbent is an exothermic process. This observation is consistent with the experimentally observed decline in adsorption capacity as temperature increases. The negative Δ*S*° (−21.08 J/mol K) indicates a decrease in randomness at the solid–solution interface, which is common in metal ion adsorption onto structured surfaces. Despite these energetically favorable indicators, all calculated Gibbs free energy values (Δ*G*°) within the temperature range of 288–313 K were found to be positive, varying from +3.05 to +3.66 kJ/mol. These positive Δ*G*° values indicate that, under the studied conditions, the adsorption process is nonspontaneous. This finding contrasts with the initial interpretation based solely on enthalpic data and suggests that, although thermodynamically feasible, the process may be hindered by kinetic barriers or require external driving forces. Thus, while Si-Carb-Formazan sorbent exhibits high affinity and adsorption capacity for Cu­(II) under optimized laboratory conditions, its thermodynamic profile does not support spontaneous adsorption at ambient or elevated temperatures without facilitation. However, it should be emphasized that the absolute Δ*G*° values obtained are relatively low (<4 kJ/mol). Overall, the adsorption may be more favorable at lower temperatures.

### 3.10 Desorption and Reusability

Metal cations such as Cu­(II) are typically desorbed using acids, bases, or chelators like Na_2_EDTA. The use of acids and bases can lead to the desorption of metal ions from the adsorbent surface due to the strong attraction of H^+^ ions and changes in charge. On the other hand, chelators like Na_2_EDTA act by forming complexes with metal ions, effectively capturing them from the surface of the adsorbent for removal. In this study, 0.1 M HNO_3_, 0.1 M HCl, and 0.1 M NaOH solutions were utilized to evaluate the effectiveness of the Si-Carb-Formazan sorbent in multiple uses. As illustrated in Figure 7a, the highest desorption rate was achieved with 0.1 M HCl. According to these results, the desorption phenomenon observed in NaOH, HNO_3_, and HCl can be interpreted more as an ion exchange-type interaction rather than purely chemical adsorption.

**Figure 7:**
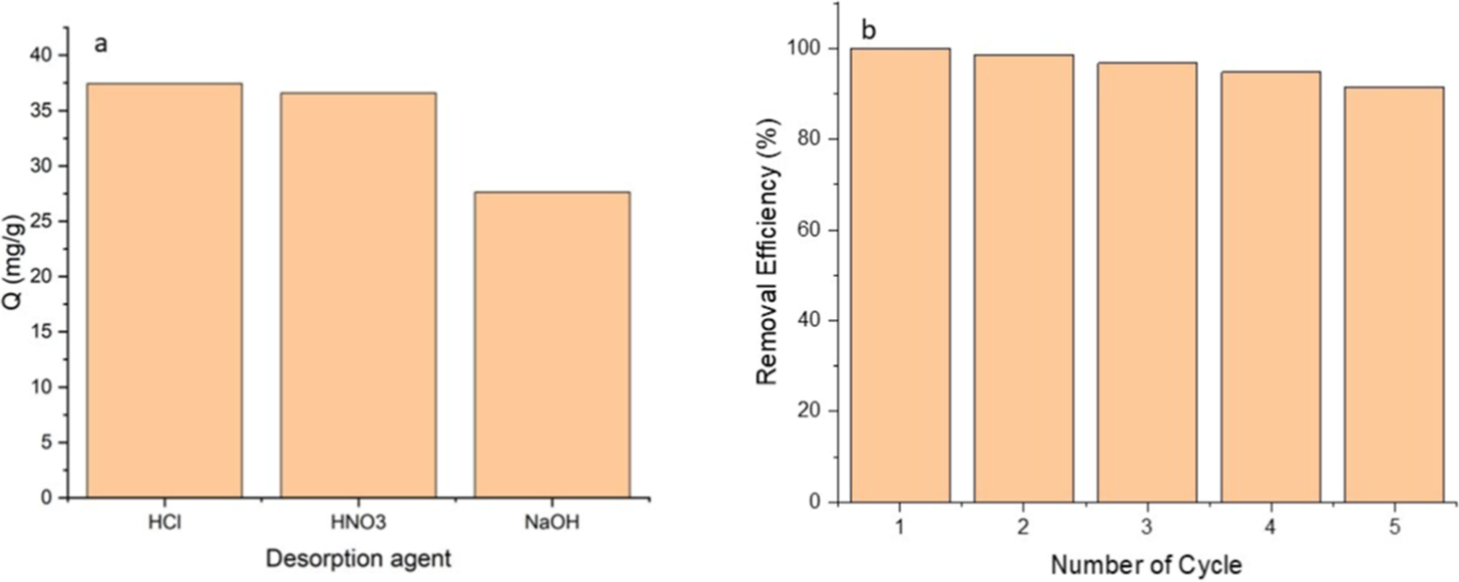
(a) Desorption of Cu from Si-Carb-Formazan; (b) reusability of the Si-carb-formazan.

Following the desorption agent’s identification, the sorbent’s reusability was assessed through repeated adsorption–desorption processes using the same sorbent over five consecutive cycles. As highlighted in Figure 7b, Cu­(II) removal efficiency utilizing the Si-Carb-Formazan sorbent diminished only 8.5% after five cycles. This slight reduction may be attributed to the loss of the sorbent during the multiple washing steps. Additionally, specific regions within the sorbent may exhibit a strong affinity for Cu­(II), which H^+^ could not entirely replace. Nevertheless, the Si-Carb-Formazan sorbent maintained its original structure without excessive swelling or degradation, confirming its reusability as an effective adsorbent for practical wastewater treatment applications.

### 3.11 Effect of Interferences

For the interference studies, mixtures with the same concentration of metals as 10 mg/L were prepared to determine the selectivity in removing Cu­(II) on the silica-formazan sorbent. The presence of Cd­(II), Zn­(II), Pb­(II), Fe­(II), and their mixtures alongside Cu­(II) as interfering ions was a deliberate choice. The selective adsorption results, as shown in Figure 8, are a testament to the strong selectivity of silica-formazan for Cu­(II). Figure 8 further illustrates this, with the Cu­(II) adsorption capacity on silica-formazan reaching 96%, a value close to the adsorption capacity in a single system. Notably, the adsorption capacity of the interfering ions was less than 90%, underscoring the robust selectivity of silica-formazan for Cu­(II).

**Figure 8:**
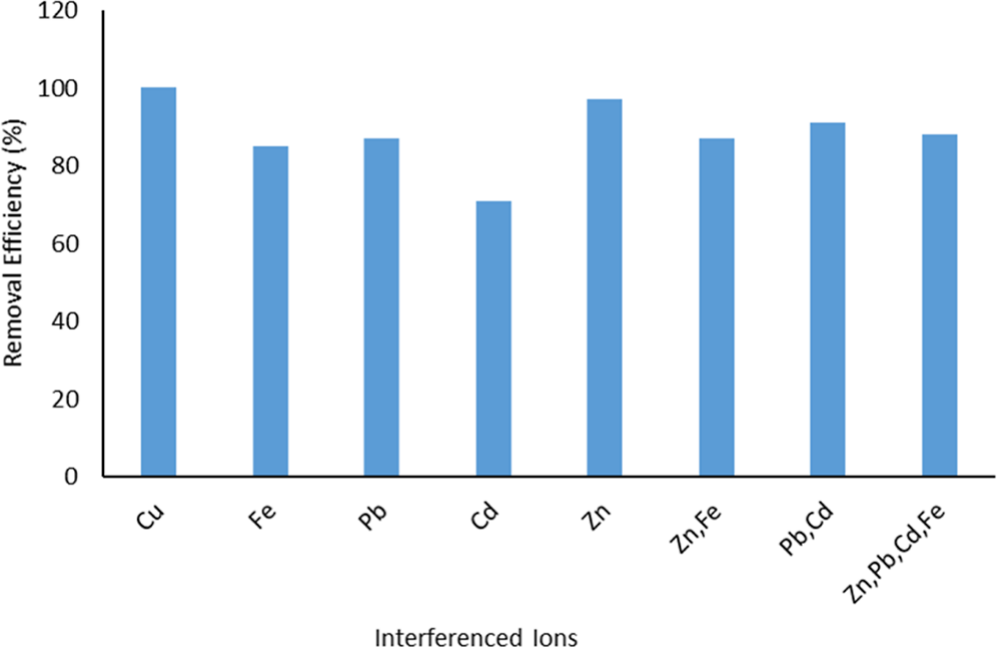
Effect of interferences.

## 4 Analysis of Real Samples

The suggested removal procedure was used to test the effectiveness of Cu­(II) removal in tap water and bottled drinking water under ideal conditions. The method’s accuracy was verified by adding 1, 2, and 5 mg/L of Cu­(II) to actual samples. Table 5 provides the recoveries along with the RSD. The recoveries demonstrated a good match between the amount of Cu­(II) removed from the examined samples and the amount added.

**Table 5:** Recoveries of Water Samples

sample	Cu(II) added(mg/L)	found (mg/L)	removal %
tap water	1.0	0.025 ± 0.006	97.5 ± 2
	2.0	0.034 ± 0.003	98.3 ± 3
	5.0	0.090 ± 0.002	98.2 ± 1
bottled drinking water	1.0	0.022 ± 0.002	97.8 ± 2
	2.0	0.052 ± 0.003	97.4 ± 4
	5.0	0.105 ± 0.005	97.9 ± 1

## 5 Comparison with Other Studies

The study shows that the Si-Carb-Formazan sorbent exhibits the highest adsorption capacity for Cu­(II) ions at 34 mg/g. For comparison, the adsorption capacities of various silica-based sorbents for Cu­(II) ions are given in Table 6. Hybrid materials have shown different potentials for the adsorption of Cu­(II). It has been reported that the adsorption efficiency on carbazole-formazan modified silica sorbent is higher or similar to most sorbents, with very little sorbent (5.0 mg) and in a very short time (15 min). Furthermore, the Si-Carb-Formazan sorbent shows promise as a suitable sorbent for removing heavy metal ions from wastewater due to advantages such as the wide surface area and structural properties conferred by its porous structure, easy functionalization, presence of chelating groups, and low cost.

**Table 6:** Cu­(II) Removal Capacity of Different Silica-Based Sorbents

metal	sorbent	contact time (min)	sorbent amount	*Q*	ref
Pb(II), Cd(II), Cu(II), Ni(II)	[NH_2_-SiO_2_MS-Ca-Alg] beads	480 (Cu(II))	15 beads	64.9 mg/g for Cu(II)	^52^
Cu(II)	CS/SA/SiO_2_ (SSC1.0)	240	0.05 g	47.50 mg/g	^47^
Cu(II)	(Si(OEt)_4_, EDTA–silane)	240	N.D	1.72 mmol/g	^53^
Pb(II), Cd(II), Cu(II), Zn(II), Mn(II)	SiO_2_ encapsulated natural zeolite (SiEZ)	60	0.5 g	186, 10.3, 12.3, 9.0, and 4.2 mg/g, respectively	^17^
Cr(III), Pb(II), Cu(II)	sago-silica composite	90	0.1 g	18.58 mg/g for Cu(II)	^54^
Cu(II)	(MCM-41-NH_2_) and (MCM-41-*N*-Hdhba)	40 and 60	50 mg	28.3 and 35.7 mg/g	^55^
Cu(II)	quaternized salicylaldehyde Schiff base modified SBA-15 mesoporous silica (SBA-AP)	240	0.04 g	34.73 mg/g	^56^
Cu(II)	Si-Carb Formazan	15	5 mg	34 mg/g	this study

## 6 Conclusion

This study presents the successful synthesis and application of a novel Si-Carb-Formazan sorbent, modified with a carbazole formazan derivative, for the efficient removal of Cu­(II) ions from aqueous solutions. The sorbent demonstrated a high adsorption capacity (34 mg/g), rapid adsorption kinetics (within 15 min), and strong selectivity for Cu­(II) ions over competing divalent metal ions, including Cd­(II), Zn­(II), and Pb­(II). The adsorption process was best described by the pseudo-second-order kinetic model and Langmuir isotherm, suggesting monolayer adsorption. Thermodynamic analysis indicated that the process is exothermic and more favorable at lower temperatures. Furthermore, the sorbent maintained its performance after five adsorption–desorption cycles, underscoring its reusability.

While the primary aim of this study was to establish a proof-of-concept for the novel application of a carbazole-formazan-based silica sorbent in Cu­(II) removal, techno-economic aspects are critical for scaling up. This study has a limitation as having relatively complex synthesis procedure of the Si-Carb-Formazan sorbent. Although the synthesis pathway involves multiple steps, it utilizes commercially available and cost-effective starting materials. Furthermore, the functionalization procedures were carried out under mild conditions, without the necessity for specialized instrumentation or energy-intensive operations. Notably, the system demonstrates a high adsorption capacity, rapid kinetics, and excellent reusability, maintaining over 90% efficiency after three regeneration cycles. These features collectively contribute to a reduced quantity of sorbent required per treatment cycle, potentially offsetting the initial synthesis expenditure. Additionally, desorption using dilute hydrochloric acid and the exclusion of toxic solvents during regeneration underscore the environmental and economic sustainability of the proposed approach. In conclusion, this study demonstrated the applicability of the Si-Carb-Formazan structure as a promising, inexpensive, and reusable sorbent for rapid adsorptive removal of Cu­(II) from wastewater.
